# Fallacious, misleading and unhelpful: The case for removing ‘systematic review’ from bioethics nomenclature

**DOI:** 10.1111/bioe.13024

**Published:** 2022-04-07

**Authors:** Giles Birchley, Jonathan Ives

**Affiliations:** ^1^ Centre for Ethics in Medicine University of Bristol, Bristol, United Kingdom of Britain and Northern Ireland

**Keywords:** concepts, empirical bioethics, methodology, systematic review

## Abstract

Attempts to conduct systematic reviews of ethical arguments in bioethics are fundamentally misguided. All areas of enquiry need thorough and informative literature reviews, and efforts to bring transparency and systematic methods to bioethics are to be welcomed. Nevertheless, the raw materials of bioethical articles are not suited to methods of systematic review. The eclecticism of philosophy may lead to suspicion of philosophical methods in bioethics. Because bioethics aims to influence medical and scientific practice it is tempting to adopt scientific language and methods. One manifestation is the increasing innovation in, and use of, systematic reviews of ethical arguments in bioethics. Yet bioethics, as a broadly philosophical area of enquiry, is unsuited to systematic review. Bioethical arguments are evaluative, so notions of quality and bias are inapplicable. Bioethical argument is conceptual rather than numerical, and the classification of concepts is itself a process of argument that cannot aspire to neutrality. Any ‘systematic review’ of ethical arguments in bioethics thus falls short of that name. Furthermore, labels matter. Although the bioethics research community may find that adopting the language and the outward methods of clinical science offers apparent prospects of credibility, policy influence and funding, we argue that such misdirection carries risks and is unlikely to pay dividends in the long term. Bioethical sources are amenable to the review methods of the social sciences, and it is on these methods that specific methods of bioethics literature review should be built.

## 1 INTRODUCTION: ECLECTICISM IN PHILOSOPHY AND THE DRIVE FOR TRANSPARENT METHODS

While bioethics is a multi‐disciplinary field of enquiry, populated by a variety of disciplines, the methods of mainstream bioethics are predominantly philosophical. Philosophical method could be described as eclectic—a process of ‘pushing and shoving’^1^ ideas to fit the argument, using ‘whatever information and whatever tools look useful’.^2^ Eclecticism itself may not be a problem, but adopting eclectic methods may ‘appear to the systematic epistemologist as a type of unscrupulous opportunis[m]’.^3^ Certainly, methodologists working in bioethics may argue that bioethical enquiry needs systematization rather than eclecticism, because non‐systematic methods produce subjective and incomplete answers to research questions. On this view, clear and transparent accounts of a bioethical process will give bioethics more credibility with clinicians and policy‐makers. Indeed, such a rationale has arguably played a part in driving the development and clear articulation of ‘empirical ethics’ methodologies,^4^ and one plank of this project has developed methods of systematic review for bioethics. It is on these methods that we focus here.

Although agreeing that bioethics should be methodologically transparent,^5^ we argue that the development of systematic review methods in bioethics *qua* systematic reviews is misguided. Some methodologists^6^ recognize that the nature of ethical argument makes it fundamentally different from the source material of much systematic review in clinical sciences. We agree, but argue that overcoming these differences with innovative bioethical methodology leaves a product that cannot usefully be described as a ‘systematic review’. After surveying the increasing popularity of systematic reviews in bioethics, we review the rationale and methods used by such reviews. We observe that methods advanced in bioethics are different from traditional systematic reviews in clinical science—by which we mean reviews that aggregate data from primary studies that measure the effectiveness of an intervention for an identified patient group—in fundamental ways. We argue that these differences invalidate the label of ‘systematic review’ in the context of bioethics, especially given the growing acknowledgement that ethical questions are unlikely to ever sensibly be answered by traditional systematic reviews. Having established that a systematic review as traditionally conceived cannot be achieved in bioethics, we then ask whether it matters if the term ‘systematic review’ is nevertheless appropriated by empirical bioethics to cover other activities (such as the aggregation of arguments or a scoping review). Although labelling a review of bioethics literature as a ‘systematic review’ could bring certain opportunities, we suggest that it also brings hazards. Most seriously, the closeness of bioethics to medicine means that the term ‘systematic review’ may mislead readers who are not familiar with bioethics methodologies. Not only is there a danger of misleading consumers of systematic reviews, but, by misidentifying bioethical methods as scientific, rather than philosophical, we ultimately risk reducing the credibility of the empirical bioethics project.

## 2 BACKGROUND

Empirical bioethics methodologies have, at least in part, been developed to counter accusations of partiality in philosophical method.^7^ Empirical bioethics methodologies are heterogenous—one review identified 32 different methods.^8^ However, an area of increasing convergence is the interest in systematic reviews of the bioethical literature.

Systematic reviews arose in clinical medicine, with a 1904 review of the effects of typhoid vaccines^9^ considered the first.^10^ Systematic reviews aim to smooth out inconsistencies between numerous single studies by aggregating their findings. Unsurprisingly, systematic review is most established in sciences replete with quantitative data that are amenable to direct comparison and combination in a metasynthesis. A systematic review is thus to be distinguished as ‘aggregating’ similar types of data to test theory, rather than as the theory‐generating processes of interpretive review characterizing the social sciences.^11^ This distinction notwithstanding, the term ‘systematic review’ is widespread beyond medicine. Examples appear in an array of areas of enquiry, including the social sciences,^12^ education^13^ and health economics.^14^ While each of these settings arouses their own debates, to maintain focus we will narrowly discuss systematic review in bioethics, beginning by assessing the quantity and methods of systematic reviews published in bioethics.

## 3 SYSTEMATIC REVIEW IN BIOETHICS

It is common^15^ to distinguish systematic reviews in bioethics that seek quantitative data pertaining to bioethical questions (e.g., ‘how many cases of euthanasia take place in cancer care in the Netherlands each year?’) from those that seek ethical answers to bioethical questions (e.g., ‘should euthanasia be allowed in cancer care in the Netherlands?’).^16^ Provided they are restricted to data that require no further interpretation, the former present no special problems to bioethics. They ask questions that can be answered with established methods of systematic review and require no special bioethical methodological innovation. Nevertheless, even in reviews that ask purely quantitative questions, the epistemically ‘thick’ nature of bioethical concepts may be problematic, so, although we will not concentrate on these types of reviews, many will inevitably be vulnerable to the criticisms we will raise.^17^ Innovation in bioethics has been focused on systematic reviews of ethical questions, and these innovative methods are the target of this article.

Many argue that adopting the methods of systematic review provides a sound methodological footing to bioethical enquiry,^18^ and interest in systematic review in bioethics is growing. Mertz, Kahrass and Strech^19^ identified 84 systematic reviews (including ‘semi‐systematic reviews’)^20^ of the ethics literature published between 1997 and 2015, with between 9 and 12 reviews being published annually in the final 4 years. Mertz, Nobile, and Kahrass^21^ added data from a further 76 bioethics reviews of empirical literature to the 84 reviews. Forty percent (65/160) of the combined sample explicitly labelled themselves as ‘systematic review’, yet only 36 of these cited specific systematic review methodologies.^22^ Since these reviews were several years old, the trends they picked up could have altered. We decided to undertake a small informal review of our own. While in no way as detailed as the studies of Mertz and colleagues, this review allowed us to become familiar with the methods currently used at first hand. We searched the PubMed database in October 2020. The search was limited to articles classified in PubMed as ‘systematic reviews’, published in the previous 5 years, that contained the keyword ‘bioethics’ in the title or abstract. A total of 122 results were screened, and we retained reviews that (i) self‐described as a systematic review in the title or abstract, and (ii) analysed an ethical question. We found 34 reviews or protocols that fitted these criteria, listed in Table 1.

**TABLE 1:** Bioethical systematic reviews and their methods

Article	*Review method described*	Method cited
Alahmad, G. (2018). Informed consent in pediatric oncology: A systematic review of qualitative literature. *Cancer Control*, *25*(1)	*Systematic search, screen and thematic analysis*	None
Aquino, Y. S., & Steinkamp, N. (2016). Borrowed beauty? Understanding identity in Asian facial cosmetic surgery. *Medicine, Health Care and Philosophy*, *19*(3), 431–441.	*Integration of narrative and systematic review*	Duke, S., & Bennett, H. (2010). Review: A narrative review of the published ethical debates in palliative care research and an assessment of their adequacy to inform research governance. *Palliative Medicine*, *24*(2), 111–126.
Barlevy, D., Elger, B. S., Wangmo, T., & Ravitsky, V. (2017). Adolescent oncofertility discussions: Recommendations from a systematic literature review. *AJOB Empirical Bioethics*, *8*(2), 106–115.	*Systematic review*	Moher, D., Liberati, A., Tetzlaff, J., Altman, D. G., & Group, P. (2009). Preferred reporting items for systematic reviews and meta‐analyses: The PRISMA statement. *PLoS Medicine*, *6*(7), e1000097.
Cooney, C. M., Siotos, C., Aston, J. W., Bello, R. J., Seal, S. M., Cooney, D. S., … & Lee, W. P. A. (2018). The ethics of hand transplantation: A systematic review. *Journal of Hand Surgery*, *43*(1), 84.e81–84.e15.	*Systematic search, screen and thematic analysis*	None
Corvol, A., Moutel, G., & Somme, D. (2016). What ethics for case managers? Literature review and discussion. *Nursing Ethics*, *23*(7), 729–742.	*Systematic reviews of empirical bioethics*	Strech, D., Synofzik, M., & Marckmann, G. (2008). Systematic reviews of empirical bioethics. *Journal of Medical Ethics*, *34*(6), 472–477.
Dewar, B., Fedyk, M., Jurkovic, L., Chevrier, S., Rodriguez, R., Kitto, S. C., Saginur, R., & Shamy, M. (2019). Protocol for a systematic scoping review of reasons given to justify the performance of randomised controlled trials. *BMJ Open*, *9*(7), e027575.	*Systematic review of reasons*	Strech, D., & Sofaer, N. (2012). How to write a systematic review of reasons. *Journal of Medical Ethics*, *38*(2), 121–126
Gómez‐Vírseda, C., de Maeseneer, Y., & Gastmans, C. (2019). Relational autonomy: What does it mean and how is it used in end‐of‐life care? A systematic review of argument‐based ethics literature. *BMC Medical Ethics*, *20*(1), 76.	*Systematic review for argument‐based clinical ethics literature*	McCullough, L. B., Coverdale, J. H., & Chervenak, F. A. (2007). Constructing a systematic review for argument‐based clinical ethics literature: The example of concealed medications. *Journal of Medicine and Philosophy*, *32*(1), 65–76.
Hofmann, B. (2017). Ethical issues with colorectal cancer screening – A systematic review. *Journal of Evaluation in Clinical Practice*, *23*(3), 631–641.	*Ethical analysis*	Bakke Lysdahl, K., & Droste, S. (2017). *Ethical analysis*. http://vortal.htai.org/index.php?q=node/11
Kalkman, S., Mostert, M., Gerlinger, C., van Delden, J. J. M., & van Thiel, G. (2019). Responsible data sharing in international health research: A systematic review of principles and norms. *BMC Medical Ethics*, *20*(1), 21.	*Systematic search, screen and thematic analysis*	None
Karpowicz, L., Bell, E., & Racine, E. (2016). Ethics oversight mechanisms for surgical innovation: A systematic and comparative review of arguments. *Journal of Empirical Research on Human Research Ethics*, *11*(2), 135–164.	*Systematic review of reasons*	Strech, D., & Sofaer, N. (2012). How to write a systematic review of reasons. *Journal of Medical Ethics*, *38*(2), 121–126.
Leider, J. P., DeBruin, D., Reynolds, N., Koch, A., & Seaberg, J. (2017). Ethical guidance for disaster response, specifically around crisis standards of care: A systematic review. *American Journal of Public Health*, *107*(9), e1–e9.	*Systematic search, screen and thematic analysis*	None
Leslie, L., Cherry, R. F., Mulla, A., Abbott, J., Furfari, K., Glover, J. J., Harnke, B., & Wynia, M. K. (2016). Domains of quality for clinical ethics case consultation: A mixed‐method systematic review. *Systematic Reviews*, *5*, 95.	*Scoping review*	Arksey, H., & O'Malley, L. (2005). Scoping studies: Towards a methodological framework. *International Journal of Social Research Methodology*, *8*(1), 19–32.
Martins Pereira, S., & Hernández‐Marrero, P. (2018). Ethical challenges of outcome measurement in palliative care clinical practice: A systematic review of systematic reviews. *Annals of Palliative Medicine*, *7*(Suppl 3), S207–S218.	*Review of reviews*	Whitlock, E. P., Lin, J. S., Chou, R., Shekelle, P., & Robinson, K. A. (2008). Using existing systematic reviews in complex systematic reviews. *Annals of Internal Medicine*, *148*(10), 776–782.
Mezinska, S., Kakuk, P., Mijaljica, G., Waligóra, M., & O'Mathúna, D. P. (2016). Research in disaster settings: A systematic qualitative review of ethical guidelines. *BMC Medical Ethics*, *17*(1), 62.	*Systematic search, screen and qualitative analysis with constant comparative method*	None
Michalsen, A., Long, A. C., DeKeyser Ganz, F., White, D. B., Jensen, H. I., Metaxa, V., … & Curtis, J. R. (2019). Interprofessional shared decision‐making in the ICU: A systematic review and recommendations from an expert panel. *Critical Care Medicine*, *47*(9), 1258–1266.	*Systematic search, screen and descriptive data extraction*	None
Mitrović, V. L., O'Mathúna, D. P., & Nola, I. A. (2019). Ethics and floods: A systematic review. *Disaster Medicine and Public Health Preparation*, *13*(4), 817–828.	*Systematic search, screen and thematic analysis*	None
Morley, G., Ives, J., Bradbury‐Jones, C., & Irvine, F. (2019). What is ‘moral distress’? A narrative synthesis of the literature. *Nursing Ethics*, *26*(3), 646–662.	*Systematic reviews of empirical bioethics; narrative synthesis of systematic review*	Strech, D., Synofzik, M., & Marckmann, G. (2008). Systematic reviews of empirical bioethics. *Journal of Medical Ethics*, *34*(6), 472–477.
Ngaage, L. M., Ike, S., Elegbede, A., Vercler, C. J., Gebran, S., Liang, F., … & Rasko, Y. M. (2020). The changing paradigm of ethics in uterus transplantation: A systematic review. *Transplant International*, *33*(3), 260–269.	*Systematic search, screen and thematic analysis*	None
Nicolini, M. E., Kim, S. Y. H., Churchill, M. E., & Gastmans, C. (2020). Should euthanasia and assisted suicide for psychiatric disorders be permitted? A systematic review of reasons. *Psychological Medicine*, *50*(8), 1241–1256.	*Systematic review of reasons*	Strech, D., & Sofaer, N. (2012). How to write a systematic review of reasons. *Journal of Medical Ethics*, *38*(2), 121–126.
Owoc, M. S., Kozin, E. D., Remenschneider, A., Duarte, M. J., Hight, A. E., Clay, M., … & Briggs, S. (2018). Medical and bioethical considerations in elective cochlear implant array removal. *Journal of Medical Ethics*, *44*(3), 174–179.	*Systematic review*	None
Padela, A. I., & Aparicio, M. K. (2019). Genethics and human reproduction: Religious perspectives in the academic bioethics literature. *New Bioethics*, *25*(2), 153–171.	*Systematic search, screen and critical discourse analysis*	None
Paul, M., O'Hara, L., Tah, P., Street, C., Maras, A., Ouakil, D. P., … & MILESTONE Consortium. (2018). A systematic review of the literature on ethical aspects of transitional care between child‐ and adult‐orientated health services. *BMC Medical Ethics*, *19*(1), 73.	*Hybrid bioethics methodology of all review types described by McDougall, R. (2014)*	McDougall, R. (2014). Systematic reviews in bioethics: Types, challenges, and value. *Journal of Medicine and Philosophy*, *39*(1), 89–97.
Perez‐Bret, E., Altisent, R., & Rocafort, J. (2016). Definition of compassion in healthcare: A systematic literature review. *International Journal of Palliative Nursing*, *22*(12), 599–606.	*Systematic search, screen, ‘expert consultation’*	None
Piasecki, J., Waligora, M., & Dranseika, V. (2017). What do ethical guidelines for epidemiology say about an ethics review? A qualitative systematic review. *Science and Engineering Ethics*, *23*(3), 743–768.	*Systematic search, screen and qualitative analysis with constant comparative method*	None
Rach, C., Lukas, J., Müller, R., Sendler, M., Simon, P., & Salloch, S. (2020). Involving patient groups in drug research: A systematic review of reasons. *Patient Preference and Adherence*, *14*, 587–597.	*Systematic review of reasons*	Strech, D., & Sofaer, N. (2012). How to write a systematic review of reasons. *Journal of Medical Ethics*, *38*(2), 121–126.
Rodrigues, P., Crokaert, J., & Gastmans, C. (2018). Palliative sedation for existential suffering: A systematic review of argument‐based ethics literature. *Journal of Pain and Symptom Management*, *55*(6), 1577–1590.	*Systematic review for argument‐based clinical ethics literature*	McCullough, L. B., Coverdale, J. H., & Chervenak, F. A. (2007). Constructing a systematic review for argument‐based clinical ethics literature: The example of concealed medications. *Journal of Medicine and Philosophy*, *32*(1), 65–76.
Rodríguez‐Prat, A., Balaguer, A., Crespo, I., & Monforte‐Royo, C. (2019). Feeling like a burden to others and the wish to hasten death in patients with advanced illness: A systematic review. *Bioethics*, *33*(4), 411–420.	*Systematic search, screen and analysis using meta‐ethnography*	None
Roest, B., Trappenburg, M., & Leget, C. (2019). The involvement of family in the Dutch practice of euthanasia and physician assisted suicide: A systematic mixed studies review. *BMC Medical Ethics*, *20*(1), 23.	*Systematic search, screen and thematic analysis*	None
Schnall, J., Hayden, D., & Wilkinson, D. (2019). Newborns in crisis: An outline of neonatal ethical dilemmas in humanitarian medicine. *Developing World Bioethics*, *19*(4), 196–205.	*Systematic search and screen*	(An appendix with detailed methods could not be located)
Schofield, G., Brangan, E., Dittborn, M., Huxtable, R., & Selman, L. (2019). Real‐world ethics in palliative care: Protocol for a systematic review of the ethical challenges reported by specialist palliative care practitioners in their clinical practice. *BMJ Open*, *9*(5), e028480.	*Systematic reviews of empirical bioethics*	Strech, D., Synofzik, M., & Marckmann, G. (2008). Systematic reviews of empirical bioethics. *Journal of Medical Ethics*, *34*(6), 472–477.
Smedinga, M., Tromp, K., Schermer, M. H. N., & Richard, E. (2018). Ethical arguments concerning the use of Alzheimer's disease biomarkers in individuals with no or mild cognitive impairment: A systematic review and framework for discussion. *Journal of Alzheimers Disease*, *66*(4), 1309–1322.	*Systematic review of reasons*	Strech, D., & Sofaer, N. (2012). How to write a systematic review of reasons. *Journal of Medical Ethics*, *38*(2), 121–126.
Sulzer, S. H., Feinstein, N. W., & Wendland, C. L. (2016). Assessing empathy development in medical education: A systematic review. *Medical Education*, *50*(3), 300–310.	*Systematic search, screen and descriptive data extraction*	None
van Dijke, I., Bosch, L., Bredenoord, A. L., Cornel, M., Repping, S., & Hendriks, S. (2018). The ethics of clinical applications of germline genome modification: A systematic review of reasons. *Human Reproduction*, *33*(9), 1777–1796.	*Systematic review of reasons*	Strech, D., & Sofaer, N. (2012). How to write a systematic review of reasons. *Journal of Medical Ethics*, *38*(2), 121–126.
Wright, D. K., Gastmans, C., Vandyk, A., & de Casterlé, B. D. (2020). Moral identity and palliative sedation: A systematic review of normative nursing literature. *Nursing Ethics*, *27*(3), 868–886.	*Systematic search, screen and qualitative data analysis*	None

As Table 1 shows (middle column), many sources described review methods with a structured search, where (i) keywords guided; (ii) searches in specified databases, whose results were; (iii) screened with specified criteria, identifying references that were; (iv) qualitatively analysed or (in the case of descriptive data) aggregated. Sixteen sources followed no cited method. One cited ‘review of reviews’^23^ and another ‘scoping review’.^24^ Another cited a method of narrative review that adopted some of the processes of systematic review.^25^ Importantly, none of them cited methods that the cited source described as ‘systematic review’, so these methods will therefore not be scrutinized here. We also will not critically examine the preferred reporting items for systematic reviews and meta‐analyses (PRISMA) systematic review guidelines, cited by a further source,^26^ as these are guidelines for aggregative review.^27^ One source^28^ cited a review of bioethics review methods,^29^ specifying that it hybridized the review described—this was surely a mistake, given that the review described different methods for seeking to answer quantitative or ethical questions. Remaining sources used methods of systematic review explicitly innovated for reviewing bioethical literature (Table 2).

**TABLE 2:** Systematic review methods in bioethics

Method and citation	Description
*Systematic review for argument‐based clinical ethics literature*: McCullough, L. B., Coverdale, J. H., & Chervenak, F. A. (2007). Constructing a systematic review for argument‐based clinical ethics literature: The example of concealed medications. *Journal of Medicine and Philosophy*, *32*(1), 65–76.	A method of systematic review of ethical arguments based on the accepted standards for reviews of medical literature. The method involves a focused question based on the identification of the patient group, intervention, comparator and outcome (PICO), a structured database search and screen, and the identification of the study conclusions, including a five‐point assessment of the quality of the arguments deployed (ranging from ‘mere opinion’ to ‘full ethical analysis’).
*Ethical analysis*: Bakke Lysdahl, K., & Droste, S. (2017). *Ethical analysis*. http://vortal.htai.org/index.php?q=node/11	A framework for systematic review that specifies a method for searching for literature (based on devising a focused question using PICO). The method contains no details of how to conduct the analysis.
*Systematic review of empirical bioethics literature*: Strech, D., Synofzik, M., & Marckmann, G. (2008). Systematic reviews of empirical bioethics. *Journal of Medical Ethics, 34*(6), 472–477.	A systematic review method for aggregating survey and interview research on ethical issues. The focus of the review is to aggregate data rather than summarize argument. The method formulates a review question based on methodology, issues and participants (MIP), and specifies methods for diversifying the sources of literature, introducing flexibility in search terms and algorithms and increasing the rigour in screening process, quality assessment and data analysis.
*Systematic review of reasons*: Strech, D., & Sofaer, N. (2012). How to write a systematic review of reasons. *Journal of Medical Ethics*, *38*(2), 121–126.	A method aimed at aggregating different arguments. Rejecting the possibility of adequately answering an ethical question with a systematic review, the authors argue that the question guiding a systematic review must be empirical. The method thus aims to summarize the different arguments encountered rather than providing a single conclusion. The method requires a research question asking which reasons have been given for/against an evaluative question, a rigorous and comprehensive search strategy that will retrieve all available literature pertaining to the question from multiple fields of enquiry, and analysis that identifies narrow and broad reasons using eight deductive categories. These, for example, identify the argument, the attitude taken towards it by the author, and the argument's alleged implications. Complex arguments are broken down into two or more broad reasons. The end result is a list of broad and narrow reasons, with numbers of occurrences quantified, from which policy‐makers can make their own evaluations.

### 3.1 Critiquing current methods

Our analysis suggests that systematic reviews in bioethics sometimes adopt methods from other areas of enquiry, or, more often, adopt a bespoke approach. In both cases the epithet of ‘systematic review’ is adopted to denote any review conducted in a transparent way. Clear accounts of method are undoubtedly important, including in bioethics. However, adopting the term ‘systematic review’ to describe methodological transparency alone seems highly problematic. Systematic review is a well described process within the clinical sciences involving distinct steps:

(i) formulating a research question;
(ii) systematically gathering resources that may answer the question;
(iii) assessing these sources for bias and validity based on their method and quality;
(iv) aggregation and presentation of data to answer the research question.^30^

These four steps define systematic review in the clinical sciences, but are not always present in systematic review methods explicitly innovated for use in bioethics (we will explore the reasons later). For example, Bakke and colleagues^31^ describe developing focused review questions and the systematic gathering of resources, but offer no method for validity assessment and aggregation. It is clearly questionable if this method should be described as ‘systematic review’. Other methods do offer innovations to address validity and aggregation. However, once we consider the divergences and weaknesses of these approaches, we argue that validity assessment and aggregation cannot be credibly achieved in reviews of ethical arguments.

#### 3.1.1 Validity assessment and quality appraisal

McCullough, Coverdale and Chervenak^32^ address the third step of assessing quality and validity through a bespoke five‐point scale. The scale has been criticized as unvalidated and of debateable usefulness.^33^ The authors base their scale upon earlier work^34^ that assesses the quality of ethical argument by applying external criteria, such as whether it uses established concepts like ‘reflective equilibrium’, the ‘four principles’^35^ or professional virtues. Yet these concepts are not measures of quality, but independent approaches to making ethical judgements that are controversial in themselves.^36^ Indeed, McCullough, Coverdale and Chervenak^37^ unwittingly attest to the difficulties in assessing quality in bioethical argument by complaining that the language of some bioethical arguments was ‘freighted with the potential for bias’.^38^ In our view, ethics literature (*qua* ethics literature) describes and argues for specific positions or beliefs held by the authors. Good arguments may be balanced, but will necessarily be partial (including, of course, the argument we make in this article). It is a fundamental mistake to consider that ethical arguments can avoid being ‘biased’, and this issue goes far beyond mere conscious preference for one argument or another.^39^ There are good grounds to argue that descriptive language and argument are by their nature evaluative,^40^ and even ‘mere’ description reflects the ‘biases’ of the author.

Strech and colleagues^41^ readily acknowledge scepticism that quality assessment of qualitative data (such as philosophical argument) is possible in a way that determines external validity. While the they opt to use one of the many methods of quality appraisal, this does not close the question of whether there is any utility in performing such an appraisal. Dixon‐Woods and colleagues^42^ had six reviewers each use three quality appraisal tools on a sample of qualitative studies. Different tools changed reviewers unprompted judgements of quality, but not always in ways that reviewers felt adequately reflected the insightfulness of the studies they were reviewing. More troubling still, there were substantial variations between reviewers' judgements of quality irrespective of the tool used. In the absence of robust measures of quality there may be a tendency to view arguments that one agrees with as ‘better quality’, simply because we are inclined to find problems with arguments we disagree with.^43^ As such, quality assessment in reviews of bioethics literature—at the point of inclusion—risks introducing the bias that systematization putatively avoids.

In a discussion of approaches to quality appraisal in bioethics, Mertz^44^ acknowledges the weaknesses of all existing methods. He attempts to make some headway by matching the different aims of reviews to appropriate ways of reporting quality, using (1) checklists, (2) ‘quality assurance criteria’ like peer review, and (3) ‘content related criteria’ such as checking the logical validity of syllogistic argument. Mertz argues that only ‘content related criteria’ have the potential to be developed into quality criteria. We agree with Mertz's assessment of how difficult such a task may be (given fundamental disagreements about concepts like argumentative logic and truth value).^45^ We observe, further, that using argumentative tools to evaluate the quality of arguments essentially flattens the distinction between quality assessment and mounting a critical argument. We are sceptical that removing arguments judged to be of poor quality in this way would not itself fatally undermine the transparency of the review process. Basing the quality of argument *only* on content related criteria is likely to put a review on one side or other of an argument before it even begins. Even if quality were measurable in this or some other way, whether poor‐quality arguments should be excluded from bioethical review by any means is questionable. Arguments that ignore relevant factors, present an unbalanced picture, or present straw man arguments may well be ‘poor quality’ in an interesting and relevant way. Although we may not ultimately accept such arguments, rejecting them should be as a result of the bioethical analysis. Excluding them from the review on the basis of poor quality pre‐empts and weakens the analysis that the review provides material for.^46^ As it is, although worthwhile, Mertz's argument for content related quality assessment at best seems to imply a merging of the quality assessment and presentation steps of systematic review. Since the intention of the quality assessment step is to exclude poor‐quality sources, we suggest that Mertz's proposed solution *de facto* removes this distinctive step of systematic review, which would make the nomenclature of ‘systematic review’ even more inappropriate.

#### 3.1.2 Aggregation data and presenting the answer

Although they offer an answer to their research question, McCullough, Coverdale, and Chervenak^47^ offer no methodological detail of how the fourth, aggregative step takes place in their method (their worked example appears to be a narrative review). Strech, Synofzik, and Marckmann^48^ aggregate data from different methods of survey research. This approach makes quantitative aggregation difficult, and the authors use qualitative analysis to aggregate these sources. This is a different process from aggregating quantitative data because qualitative methods explicitly rely on evaluations of the reviewer. The qualitative paradigm recognizes researcher subjectivity, so regards reviewer evaluations as unproblematic. However, this positions the aggregative method outside the objective paradigm—most commonly associated with the natural sciences, but widely adopted in clinical science—that underlies quantitative systematic review. Since approaches using qualitative methods are different from ‘hard science’ approaches to systematic review, it is unclear what is gained by describing a qualitative review as a ‘systematic review’ rather than as some form of qualitative meta‐analysis.

Aggregating qualitative data—for example, philosophical arguments—and drawing conclusions requires judgements about meaning and value. The genius of the ‘systematic review of reasons’ proposed by Strech and Sofaer^49^ is that it addresses this problem by attempting to remove evaluation from the reviewer's purview, instead presenting these evaluative choices to the review's (policy‐orientated) readers to make. Ostensibly, attempting to objectively present the range of arguments found in the literature and to leave it to the reader to judge which is best appears convincing. Nevertheless, the first—perhaps trivial—downside is that removing this final stage from aggregation diverges quite significantly from a ‘systematic review’ as understood in clinical science. Less trivially, the method of aggregation does not, in fact, remove the evaluative judgements of the reviewer, but just pushes them down one level. This is because the ‘systematic review of reasons’ asks reviewers to group similar reasons together. Reviewers therefore need to evaluate whether the similarities between different reasons are enough to justify such grouping, or whether the reasons warrant new categories. Once a category is decided upon, further evaluations will be needed to describe the groups of reasons. This is an evaluative process: ‘[t]o describe is to take a stance’.^50^

Readers might be sceptical about the degree of evaluation that we suggest is necessary to categorize and describe reasons. Arguably, clinical science is also susceptible to this need for evaluation, and so a conventional clinical systematic review is not value‐free in a strong sense. Moreover, if systematic reviewers are experienced bioethicists, they will have little problem classifying different sets of reasons in line with wider theory: for example, deciding if reasons are autonomy‐supporting or coercion‐supporting may be a rudimentary interpretive task. More must therefore be said about the difficulties involved.

### 3.2 The aggregation of data is not value‐free

No systematic review is completely objective.^51^ Judgement calls are ubiquitous in systematic reviews of clinical science. For example, a comparison of two contemporaneous clinical systematic reviews found discrepancies between the articles selected and between the conclusions drawn by each review team.^52^ Despite apparent defects, it is arguable that the aggregative stage of a scientific review, which ideally compares data from similar, validated measures and accounts for operator variability, can produce data that are ‘objective’ within the common scientific understanding of objectivity.

The latitude of judgement needed in a bioethics review may be wider than in clinical science. We categorize reasons according to how we conceptualize these categories, and conceptualization of complex reasons is not a precise science. Bioethics researchers do categorize and aggregate reasons every time they use bioethical arguments or concepts. Yet this process is necessarily—and unashamedly—evaluative. An attempt to neutrally group or describe sets of reasons encounters many problems related to conceptualization.

There are tacit signs that the innovators of the ‘systematic review of reasons’ are aware of the obstacle that conceptual evaluation presents, because they note that numerous key concepts within bioethical discussions are used differently in different research articles.^53^ To some extent, this problem is part of the fabric of bioethics, because some bioethical concepts arise from attempts to accommodate value pluralism. Ethical concepts like ‘autonomy’ are arguably intentionally vague so that a plurality of agents can subscribe to them whatever their individual values. Although difficulties in classifying concepts may be reduced by having multiple systematic reviewers seek inter‐rater agreement, as researchers investigating quality evaluation found,^54^ it can be challenging to find common ground.

Attempts to develop definitive concepts fail to appreciate advances in the understanding of concepts.^55^ One obstacle is the variety of ‘typicality effects’ that concepts are subject to. Experiments indicate that raters judge some instances of a concept more typical of that concept than others. For example, the concept ‘fruit’ is more quickly and consistently associated with ‘apples’ than with ‘olives’.^56^ Worse, some concepts—Machery^57^ suggests the example of ‘heaps’—have many instances where individual judgements of typicality show little stability. In other words, on consecutive occasions we may change our opinion of whether a given pile of things counts as a ‘heap’ or not. Others have also argued that some classes of complex concepts have no typical instances at all,^58^ so there is little chance of classifying them consistently. It may therefore be hard to agree that certain reasons belong to certain classes. Not only this, but the very act of classification may be intimately tied to moral evaluation. Experimenters who asked participants to classify behaviours that result in the death of a foetus as ‘killing’ or ‘letting die’ found that the participants’ classifications were made according to their antecedent moral beliefs.^59^ It is credible to argue that classifications of complex concepts—like ethical arguments—into categories are innately open to interpretation and contention. Although we resist the conclusion that people cannot agree about any values because they suffer radical epistemic individualism,^60^ we do suggest that when it comes to bioethical concepts, reaching agreement is an intensive job that requires plenty of explanation and argumentative support. This processis certainly not capable of providing the sort of neutral, definitive classification of reasons for the consumption of decision‐makers that is envisaged in the ‘systematic review of reasons’.

In sum, the goals of systematic reviews in clinical science seem impossible to achieve in bioethics. All bioethical systematic review methods involve significant departures from the steps of scientific systematic review. Bioethical methods either avoid assessing for bias and quality or providing an answer, or tackle these steps with methods that are unsuited to the task.

In answer, defenders of systematic reviews in bioethics may protest that these methods have resulted in some interesting and informative reviews, so none of this matters. While agreeing with the former, we disagree with the latter: describing bioethical reviews as ‘systematic reviews’ has consequences. We shall therefore explore what is gained and risked by adopting the label of ‘systematic review’. We will argue that, on balance, doing so creates false expectations of bioethical research and risks the credibility of bioethical enquiry.

## 4 LABELS AND CONSEQUENCES

Literature reviews are important in all fields of enquiry, but there are many ways of accomplishing reviews. The adoption of the specific label of ‘systematic review’ for reviews in bioethics, despite the technical divergence of these reviews from systematic review in clinical science, putatively increases the potential for policy influence by asserting an authoritative scientific identity for bioethics. In this section we set out what is gained and risked by pursuing this agenda.

### 4.1 Influencing policy

The huge proliferation of research articles—1,754,932 new articles were indexed in 2021 in PubMed alone^61^ —challenges researchers of all disciplines seeking a comprehensive understanding of their field. Reviews give efficient access to this growing knowledge. Systematic reviews are particularly valuable because they promise to capture the entirety of knowledge about a particular enquiry. While this is useful to topic experts, it is even more useful to those outside the field. By capturing all arguments and literature, systematic review in bioethics promises policy influence.^62^ It does so by providing clinicians and policy‐makers with a way to quickly grasp the scope of bioethical arguments in a digestible, trusted and familiar form. Influencing policy is not just personally satisfying, it is career‐enhancing: academic policy is driven partly by claims that academic activity is worthwhile if it can demonstrate value for money.^63^ By giving their activities a way to impact upon policy, bioethicists may find that conducting systematic reviews is existentially important.

Conducting reviews to inform policy‐makers is a legitimate and sensible activity (so long as it can be established that the reviews actually reach this audience). Nevertheless, because complex concepts are inherently subjective, bioethics reviews are—and we would argue must always be—partial and incomplete appraisals of the topic. Policy‐makers familiar with scientific systematic reviews may misunderstand bioethical ‘systematic review’ to be the last word on an ethical question. Of the methods we have identified, only one^64^ takes steps to mitigate this problem by ostensibly avoiding evaluative conclusions. We question whether it is possible to avoid evaluation, but even if it is, simply listing summary arguments may arguably produce less reasoned policy.

Removing evaluations will lead policy‐makers to make their own judgement about which arguments are better or worse, but on what basis? Policy‐makers may make inferences about ethical validity from other data, for example how frequently arguments are repeated. We may warn policy‐makers not to make such inferences,^65^ but for warnings to be effective, consumers must be competent to follow them. Unfortunately, this is unrealistic. Summary arguments will advocate particular positions, but cannot expose flaws in opposing views, and this creates an obstacle to weighing up the merits of different arguments. In situations of information deficiency, reasoners use heuristic approaches where they intuit differences based on unconscious biases^66^ or on a single, easily available factor^67^ (like frequency). Removing a reviewer's evaluation of arguments in favour of a ‘neutral’ description may, paradoxically, make policy less well reasoned. In fact, a better approach would be both to allow reviewers to make evaluations and to stop calling these reviews ‘systematic reviews’. If they wished, policy‐makers could consult several such subjective evaluations of the evidence. Doing so would be a more honest way to approach (and present) the evidence, and would result in more reasoned policy.

### 4.2 Scientific authority

It is true that ‘systematic reviews have a special authority for clinical decision‐makers and policymakers’.^68^ Bioethics addresses clinicians and scientists with action‐guiding norms. Proposing these through systematic review suggests that these norms are valid in a way that clinicians and scientists recognize and respect. Thus, systematic review serves to validate a view of bioethics as possessing quasi‐scientific authority. We would not impute the motives of those who choose a systematic review methodology—indeed, we have tried such methods ourselves. There will be many who simply feel that systematic reviews are a valuable tool for identifying, collating and presenting information (and we note that we do not dispute the value of such work, only the chosen nomenclature and associated risks). Nevertheless, scientific authority may be important for bioethics as a discipline for several reasons, including *purpose*, *funding*, and clinical *credibility*.

One *purpose* of bioethics is predicated on the idea that good science (often, but not always, clinical science) must reflect on the ethical implications of scientific activities. Although bioethics could scrutinize science from the outside, it is arguably more influential when embedded in scientific or clinical practice. Such a view may have existentially important impacts on bioethics funding. Positioning bioethics as an element of the scientific process may help bioethics to appeal to a certain mindset within policy‐making and *funding* that places special value on ‘scientific’ paradigms.^69^ Adapting the tools of scientific enquiry to bioethics, as the adoption of ‘systematic reviews’ does, helps in the pitching of bioethical studies as partners of science and technology research, placing bioethics funding (at least in theory) on a more secure footing. Finally, the contention that systematic reviews have a special authority reflects a plausible, widespread view that systematic reviews command a special clinical *credibility*, and such credibility would help bioethics to serve its purpose.

Of course, we can trivially note that science funding is neither reliably generous nor immune from political fashions.^70^ However, a more important observation is that a relationship with science does not and should not allow bioethics to lay claim to a sort of scientific authority. Science should be informed of the implications of bioethical enquiry, and part of this work can be done by conducting bioethics projects in tandem with scientific ones. Bioethics should therefore be a part of conducting good science, but not because bioethics *is* a science.

Positioning bioethics towards science may in fact present risks to the reputation of bioethics as an area of inquiry. Bioethics must be clear that what it and science can achieve are different. Much of clinical science aspires to aggregate data that give confident answers to clinical problems. While it is thus necessary for systematic reviews in clinical science to be both limited and updated to account for societal factors, bioethical answers to ethical problems are even more reliant on socially and politically contingent norms and values. Systematic reviews in bioethics might avoid this problem by giving clear statements that they are limited in this way, and by strictly focusing on individual cultures and jurisdictions. However, they might more clearly avoid misinterpretation by adopting methodological labels and approaches that highlight the differences between bioethical and clinical findings. From this point of view, it seems likely that the term ‘systematic review’ will mislead non‐bioethicists. Consumers of systematic reviews will include those who seek accessible information about areas outside their field, who will concentrate on headline findings and are unlikely to look beyond methodological labels or scrutinize differences in approach. Because ‘systematic review’ carries a particular scientific cachet that transcends disciplinary boundaries, systematic reviews in bioethics seem aimed to command attention above that of run of the mill bioethical discourse, and may thus attract serious misunderstanding.

We recognize that without requisite evidence of the reach of systematic reviews in bioethics, both the above claims are speculative. We know of no compelling evidence that policy‐makers routinely read systematic reviews of bioethics, much less respond to them. However, we do believe that there are sufficient grounds for concern that the cachet of systematic review could be taken by clinicians, bioethicists and researchers in other disciplines to indicate broad consensus in bioethics when no such consensus exists. Citation tracking of a well‐cited bioethical systematic review on an issue with which we have familiarity, conducted by an experienced methodologist, shows that systematic review is cited in this way in bioethical, legal and clinical journals.^71^ In our view, this is despite wider debate indicating little consensus on the issue it reviews within bioethics.^72^ While we accept that there may be unique reasons for way the review is cited in this case (the authors made specific claims of consensus in their review) and that a more robust approach must be taken to determining the broader impact of bioethical systematic review to reach a more definitive view, we think it shows grounds to worry about a pernicious effect of using the label of ‘systematic review’.^73^

#### 4.2.1 A better approach

We have disputed the claim that bioethics needs systematic reviews *qua* systematic reviews. Whatever the advantages, given that systematic reviews do not more adequately inform policy and may encourage the misrepresentation of bioethics *as* clinical science, we believe there is questionable utility in using the terminology of ‘systematic reviews’ at all. Indeed, since many of the methods of bioethical ‘systematic review’ reviewed above use broadly qualitative approaches, it is not clear why bioethics should resist identifying with methods of qualitative meta‐analysis. Systematic reviews properly aim to identify and aggregate data in order to provide a definitive answer to a clearly defined and specific question. A literature review in bioethics cannot do this. Bioethics reviews must inevitably concede the goal of providing a definitive, aggregated answer because bioethical arguments are not a zero‐sum game where a correct answer (where ‘correct’ refers to either a ‘correct ethical conclusion’ or a ‘correct description of the issues or arguments’) can be divorced from the viewpoint of the reviewer. As we acknowledge, some methodologists do concede this. But this is presented as an adaption that allows the concept of systematic review to be transferred to bioethics. We suggest that the concept of systematic review, which bolts inherent connotations of objectivity onto a transparent review process, is not well suited to bioethics in any form.

We suggest that the methods of the social sciences are ultimately a better model on which to base bioethics review methodologies than the methods of the clinical sciences, given the sorts of enquiries that bioethics undertakes. In comparison with social science literature, bioethics may contain more purely philosophical work and less primary empirical data (but is surely as reliant on empirical claims) and be more concerned with meta‐ethical issues. Nevertheless, both present their findings in substantially similar terms. This does not mean that a bioethics review must slavishly follow the methods of social science, more that these methods provide a good foundation on which to develop our own.

We do not intend to review approaches from social science here—others have already written on this topic.^74^ Instead, our central argument is that bioethics should avoid identifying review as a systematic review at all. Although we endorse eclectic approaches, we suggest that there are two characteristics that should guide bioethical reviews. Firstly, they should be transparent. This suggests using methods that systematically select a wide range of sources and guard against the well‐documented human tendency to seek arguments that justify our beliefs and to avoid those that are unfamiliar or disagreeable^75^ We acknowledge that transparency is consistent with much existing practice. Secondly, they should not pretend to neutrally present source arguments, but should be explicit in taking a critical and/or interpretative stance, supported by appropriate argument. Ethical arguments in sources may proceed by analogy and thought experiment, which require a high degree of reframing to be conveyed. An argument is needed to legibly connect different sources. Moreover, just as history shows that science has been allied to particular social orders,^76^ bioethics too is linked to established and emerging social orders.^77^ Many normative assumptions within such orders form implicit parts of arguments, and this seems an important aspect of the literature that a review should reflect. This is important because bioethical review is not a scientific review, but a philosophical review. It cannot fail to present a perspective on the review question, and should not aspire to scientific neutrality.

## 5 CONCLUSION

Philosophy has ‘not been so favoured by fate as to have been able to enter upon the secure course of a science, even though it is older than all other sciences’.^78^ While the gap between philosophy and science has steadily grown, the temptation to replicate the successes of science remains strong, especially in a science‐facing area of philosophical inquiry like bioethics. Yet replication is unsuccessful. Methodologies devised for bioethical systematic review falter because the nature of bioethical argument is intrinsically value‐based, and as such does not lend itself to assessment of quality, assessment of bias, or to neutral aggregation. Moreover, the types of data that bioethics articles deal in are conceptually complex and as such present major problems to classification and agreement. These problems should give us pause to consider whether ‘systematic review’ is a suitable label for bioethics literature reviews. Whatever the attractions of adopting the label of ‘systematic review’, it is more likely to mislead policy‐makers and misrepresent the strengths and weaknesses of bioethics, ultimately undermining the credibility of bioethical enquiry. Bioethical enquiry is closer to the social sciences than to clinical science. It is from social science methods that bioethics should build approaches to review, rather than adopting the hollow label of ‘systematic review’.

## FUNDING INFORMATION

This research was funded in whole, or in part, by the Wellcome Trust Grant No. 209841/Z/17/Z. For the purpose of Open Access, the authors have applied a CC BY public copyright licence to any Author Accepted Manuscript version arising from this submission.

## CONFLICTS OF INTEREST

The authors declare no conflicts of interest.

## Footnotes

1 Nozick, R. (1974). *Anarchy, state and utopia*. Basic Books, p. XIII.

2 Godfrey‐Smith, P. (2017). *Other minds*. HarperCollins, p. 12.

3 Einstein, A. (1949). Remarks concerning the essays brought together in this co‐operative volume. In P. A. Schilpp (Ed.), *Albert Einstein: Philosopher‐scientist* (Vol. 7, pp. 683–684). The Library of Living Philosophers.

4 Ives, J., Dunn, M., & Cribb, A. (2017). *Empirical bioethics: Theoretical and practical perspectives*. Cambridge University Press.

5 Ives, J., & Dunn, M. (2010). Who's arguing? A call for reflexivity in bioethics*. Bioethics*, *24*(5), 256–265.

6 Sofaer, N., & Strech, D. (2012). The need for systematic reviews of reasons*. Bioethics*, *26*(6), 315–328.

7 Ives et al., op. cit. note 4.

8 Davies, R., Ives, J., & Dunn, M. (2015). A systematic review of empirical bioethics methodologies*. BMC Medical Ethics*, *16*, 15.

9 Pearson, K. (1904). Report on certain enteric fever inoculation statistics*. British Medical Journal*, *3*, 1243–1246.

10 Clarke, M. (2018). Partially systematic thoughts on the history of systematic reviews*. Systematic Reviews*, *7*, 176.

11 Dixon‐Woods, M., Cavers, D., Agarwal, S., Annandale, E., Arthur, A., Harvey, J., … & Sutton, A. J. (2006). Conducting a critical interpretive synthesis of the literature on access to healthcare by vulnerable groups*. BMC Medical Research Methodology*, *6*, 35.

12 Petticrew, M., & Roberts, H. (2006). *Systematic reviews in the social sciences: A practical guide*. Blackwell.

13 Andrews, R. (2005). The place of systematic reviews in education research*. British Journal of Educational Studies*, *53*(4), 399–416.

14 Pillai, N., Dusheiko, M., Burnand, B., & Pittet, V. (2017). A systematic review of cost‐effectiveness studies comparing conventional, biological and surgical interventions for inflammatory bowel disease*. PLoS One*, *12*(10), e0185500.

15 McDougall, R. (2014). Systematic reviews in bioethics: types, challenges, and value. *Journal of Medicine and Philosophy*, *39*(1), 89–97; Sofaer & Strech, op. cit. note 6.

16 Reviews of the literature conducted in 2016 and 2020 by Mertz et al. (discussed below) developed further distinctions between the bioethics reviews they studied based on the types of ethical information the studies sought. We have not utilized these distinctions here because we are sceptical that these distinctions can be usefully mobilized. The authors distinguish between systematic reviews of the ethics literature by identifying a number of types of information that reviews claim to seek. Specifically, these include: ‘issues’, ‘reasons’, ‘principles’, ‘ethical approach' and ‘procedures’. Descriptively, we have no objection to this. However, we found the distinction between these, especially ‘issues’, ‘reasons’ and ‘principles’, to be often unclear. For example, Mertz's 2016 review gives the example of an ‘issue’ as being ‘justice in disability’. However, ‘justice’ is not *just* an issue but can meaningfully be described as a ‘concept’, and could also be a ‘reason’. Although the 2020 review makes the distinction clearer—the categories are respectively applied to examples of ethical problems in practice, arguments for and against and moral values—the distinction seems vulnerable to slippage between the three categories that Mertz's 2016 definition exemplifies (we could envisage concepts such as autonomy featuring in each). We remain sceptical that these sorts of thick epistemic descriptors can offer useful distinctions to finesse methods of bioethical review. See: Mertz, M., Kahrass, H., & Strech, D. (2016). Current state of ethics literature synthesis: a systematic review of reviews. *BMC Medicine*, *14*(1), 152; Mertz, M., Nobile, H., & Kahrass, H. (2020). Systematic reviews of empirical literature on bioethical topics: Results from a meta‐review*. Nursing Ethics, 27*(4), 960–978.

17 For example, the thick concept of ‘euthanasia’ in our example could pose problems for an aggregative review, given the many overlapping distinctions in this area between active euthanasia, passive euthanasia, physician‐led suicide, mercy killing etc. These distinctions are inevitably interpretive and thus vulnerable to some of the arguments we will later make about the pathologies of concepts and the evaluative nature of language. See: Mertz, Kahrass, & Strech, op. cit. note 16; Mertz, Nobile, & Kahrass, op. cit. note 16.

18 McCullough, L. B., Coverdale, J. H., & Chervenak, F. A. (2007). Constructing a systematic review for argument‐based clinical ethics literature: the example of concealed medications. *Journal of Medicine and Philosophy*, *32*(1), 65–76; McDougall, op. cit. note 15; Sofaer & Strech, op. cit. note 6; Strech, D., & Sofaer, N. (2012). How to write a systematic review of reasons. *Journal of Medical Ethics, 38*(2), 121–126; Strech, D., Synofzik, M., & Marckmann, G. (2008). Systematic reviews of empirical bioethics. *Journal of Medical Ethics*, *34*(6), 472–477.

19 Mertz, Kahrass, & Strech, op. cit. note 16.

20 Ibid: 2.

21 Mertz, Nobile, & Kahrass, op. cit. note 16.

22 Mertz, M., Strech, D., & Kahrass, H. (2017). What methods do reviews of normative ethics literature use for search, selection, analysis, and synthesis? In‐depth results from a systematic review of reviews*. Systematic Reviews*, *6*(1), 261.

23 Martins Pereira, S., & Hernández‐Marrero, P. (2018). Ethical challenges of outcome measurement in palliative care clinical practice: A systematic review of systematic reviews*. Annals of Palliative Medicine*, *7*(Suppl 3), S207–S218.

24 Leslie, L., Cherry, R. F., Mulla, A., Abbott, J., Furfari, K., Glover, J. J., … & Wynia, M. K. (2016). Domains of quality for clinical ethics case consultation: A mixed‐method systematic review. *Systematic Reviews, 5*, 95.

25 Duke, S., & Bennett, H. (2010). Review: A narrative review of the published ethical debates in palliative care research and an assessment of their adequacy to inform research governance. *Palliative Medicine*, *24*(2), 111–126.

26 Barlevy, D., Elger, B. S., Wangmo, T., & Ravitsky, V. (2017). Adolescent oncofertility discussions: Recommendations from a systematic literature review. *AJOB Empirical Bioethics*, *8*(2), 106–115.

27 A proposal for a bioethics orientated ‘Prisma‐Ethics’ is available as a pre‐print at the time of writing see: Kahrass, H., Borry, P., Gastmans, C., Ives, R., van der Graaf, R., Strech, D., & Mertz, M. (2021). *PRISMA‐ethics – Reporting guideline for systematic reviews on ethics literature: Development, explanations and examples*. https://osf.io/g5kfb/—we note that Prisma‐Ethics is motivated by many of the concerns we express. However, we dispute the fundamental premise of Prisma‐Ethics that we can overcome these problems and produce something that can be validly labelled as ‘systematic review’—indeed the pre‐print goes so far as to argue more reviews in bioethics should adopt this label. Given Prisma‐Ethics is not yet peer‐reviewed and published we are wary of more thorough‐going engagement at this stage, but we note its relevance to the ongoing debate.

28 Paul, M., O'Hara, L., Tah, P., Street, C., Maras, A., Ouakil, D. P., … & MILESTONE Consortium Collaborators. (2018). A systematic review of the literature on ethical aspects of transitional care between child‐ and adult‐orientated health services. *BMC Medical Ethics*, *19*(1), 73.

29 McDougall, op. cit. note 15.

30 Higgins, J., Thomas, J., Chandler, J., Cumpston, M., Li, T., Page, M., & Welch, V. (2020). *Cochrane handbook for systematic reviews of interventions, version 6.1*. Retrieved from www.training.cochrane.org/handbook

31 Bakke Lysdahl, K., & Droste, S. (2017). *Ethical analysis*. Retrieved from http://vortal.htai.org/index.php?q=node/11

32 McCullough et al., op. cit. note 18.

33 Sofaer & Strech, op. cit. note 6; Mertz, M. (2019). How to tackle the conundrum of quality appraisal in systematic reviews of normative literature/information? Analysing the problems of three possible strategies *BMC Medical Ethics, 20*, 81 (translation of a German paper).

34 McCullough, L., Coverdale, J., & Chervenak, F. (2004). Argument‐based medical ethics: A formal tool for critically appraising the normative medical ethics literature*. American Journal of Obstetrics and Gynecology*, *191*(4), 1097–1102.

35 Beauchamp, T. L., & Childress, J. F. (2009). *Principles of biomedical ethics* (7th ed.). Oxford University Press.

36 DeGrazia, D. (2003). Common morality, coherence, and the principles of biomedical ethics. *Kennedy Institute of Ethics Journal*, *13*(3), 219–230; Doris, J. (2002). *Lack of character: Personality and moral behaviour*. Cambridge University Press; Strong, C. (2010). Theoretical and practical problems with wide reflective equilibrium in bioethics*. Theoretical Medicine and Bioethics*, *31*(2), 123–140.

37 McCullough et al., op. cit. note 18.

38 Ibid: 72.

39 Bias goes deeper than mere partiality that can be corrected by conscious control. Humans have a well‐developed capacity to reconstruct narratives, including moral narratives, in self‐serving ways. There is a wealth of experimental evidence that such activity and other sources of bias are unconscious and subject to numerous, often affective, triggers. What's more, even when alerted to biases, they remain thoroughly pernicious and resistant to conscious attempts at control, especially in the medium‐long term. See, for example: Batson, C. D. (2017). Getting cynical about character: A social psychological perspective. In W. Sinnott‐Armstrong & C. B. Miller (Eds.). *Moral psychology, Vol. 5: Virtue and character* (pp. 11–44). Bradford Books; Eskine, K., Kacinic, N., & Prinz, J. (2011). A bad taste in the mouth: Gustatory influences on moral judgment. *Psychological Science*, *22*, 295–299; Kelly, D., Machery, E., & Mallon, R. (2013). Race and racial cognition. In J. Doris (Ed.), *The moral psychology handbook* (pp. 433–472). Oxford University Press.

40 Putnam, H. (2002). *The collapse of the fact/value distinction and other essays*. Harvard University Press, pp. 111–134.

41 Strech et al., op. cit. note 18.

42 Dixon‐Woods, M., Sutton, A., Shaw, R., Miller, T., Smith, J., Young, B., … & Jones, D. (2007). Appraising qualitative research for inclusion in systematic reviews: A quantitative and qualitative comparison of three methods*. Journal of Health Services Research and Policy*, *12*(1), 42–47. We acknowledge that this paper was focussed on qualitative social science data and not philosophical argument, but our position is that there are sufficient similarities between qualitative interpretation of data and qualitative argumentation (in terms of the reliance on conceptual analysis and coherence) that make the research, if not directly then indirectly, transferable.

43 Lerner, J. S., & Tetlock, P. E. (2003). Bridging individual, interpersonal, and institutional approaches to judgment and decision making: The impact of accountability on cognitive bias. In S. L. Schneider & J. Shanteau (Eds.), *Emerging perspectives on judgment and decision research* (pp. 431–457). Cambridge University Press.

44 Mertz, op. cit. note 33.

45 Rorty, R. (Ed.) (1992). *The linguistic turn*. University of Chicago Press.

46 A consequence that the light‐touch approaches to quality appraisal—or their absence altogether—in many systematic reviews in bioethics may well tacitly acknowledge.

47 McCullough et al., op. cit. note 18.

48 Strech, Synofzik, & Marckmann, op. cit. note 18.

49 Strech & Sofaer, op. cit. note 18.

50 Habermas, J. (2017). *Truth and justification*. Polity Press, p. 225.

51 Linde, K., & Willich, S. N. (2003). How objective are systematic reviews? Differences between reviews on complementary medicine*. Journal of Royal Society of Medicine*, *96*(1), 17–22.

52 Rosen, L., & Suhami, R. (2016). The art and science of study identification: A comparative analysis of two systematic reviews. *BMC Medical Research Methodology*, *16*(24).

53 Sofaer & Strech, op. cit. note 6.

54 Dixon‐Woods et al., op. cit. note 42.

55 Machery, E. (2009). *Doing without concepts*. Oxford University Press.

56 Rosch, E. (1973). On the internal structure of perceptual and semantic categories. In T. Moore (Ed.), *Cognitive development and acquisition of language* (pp. 111–144). Academic Press.

57 Machery, op. cit. note 55.

58 Murphy, G. L. (1988). Comprehending complex concepts. *Cognitive Science*, *12*, 529–562.

59 Cushman, F., Knobe, J., & Sinnott‐Armstrong, W. (2008). *Moral appraisals affect doing/allowing judgments. Cognition, 108*(1), 281–289.

60 Cherry, C. (1980). Knowing, imagining and sympathizing. *Ratio*, *22*, 133–144; Toombs, S. K. (1992). *The meaning of illness. A phenomenological account of the different perspectives of physician and patient*. Kluwer.

61 Pubmed. (2021). *Results by year*. Retrieved from https://pubmed.ncbi.nlm.nih.gov/?term=1800%3A2100%5Bdp%5D%26filter=years.2021-2021%26timeline=expanded

62 Sofaer & Strech, op. cit. note 6.

63 Bandola‐Gill, J. (2019). Between relevance and excellence? Research impact agenda and the production of policy knowledge*. Science Public Policy*, *6*(46), 895–905.

64 Strech & Sofaer, op. cit. note 18.

65 Ibid.

66 Kahneman, D., & Frederick, S. (2005). A model of heuristic judgment. In K. J. Holyoak & R. G. Morrison (Eds.), *The Cambridge handbook of thinking and reasoning* (pp. 267–293). Cambridge University Press.

67 Gigerenzer, G., Todd, P., & ABC Research Group. (1999). *Simple heuristics that make us smart*. Oxford University Press.

68 Sofaer & Strech, op. cit. note 6, p. 326.

69 For example, UK Government. (2021). *UK research and development roadmap*. Retrieved from https://www.gov.uk/government/publications/uk-research-and-development-roadmap/uk-research-and-development-roadmap

70 Campaign for Science and Engineering. (2014). *Case analysis of departmental R&D spend 2011/12*. Retrieved from https://www.sciencecampaign.org.uk/static/uploaded/1bd04de7-d627-4976-a856d42a1e905424.pdf

71 On Clarivate ‘Web of Science’ (https://www.webofscience.com/wos/woscc/basic-search), we found 43 citations for: McDougall, R., & Notini, L. (2014). Overriding parents’ medical decisions for their children: A systematic review of normative literature. *Journal of Medical Ethics*, *40*(7), 448–452. Twenty‐two of these were from *Bioethics* journals, 9 from medical journals, and 12 from other disciplines. While most cited the review without giving it specific weight, a number suggested the review's arguments had special status. These were in the following disciplines: [I] *Academic law*: Auckland, C., & Goold, I. (2020). Re‐evaluating ‘best interests’ in the wake of Raqeeb v Barts NHS Foundation Trust & Anors. *Modern Law Review*, *83*(6), 1328–1342. ‘This idea is widely supported in the ethics literature’ (p. 1388). (The sole citation for this claim is McDougall, R., & Notini, L., 2014); Auckland, C., & Goold, I. (2019). Parental rights, best interests and significant harms: Who should have the final say over a child's medical care? *Cambridge Law Journal*, *78*(2), 287–323. ‘McDougall and Notini point out from a systematic review of the ethical literature that “most bio‐ethicists argue that harm is a better threshold. There is a consensus on this issue’” (p. 312). [II] *Bioethics*: Dubin, S., Lane, M., Morrison S, Radix, A., Belkind, U., … & Inwards‐Breland, D. (2020). Medically assisted gender affirmation: When children and parents disagree. *Journal of Medical Ethics*, *46*, 295–299. ‘Consensus supports Diekema's conclusions that the harm threshold is more robust ethical guidance than “best interest” standards’ (p. 298). (Sole citation is McDougall, R., & Notini, L., 2014); Brummett, A. (2019). Whose harm? Which metaphysic? *Theoretical Medicine and Bioethics*, *40*, 43–61. ‘… the current consensus among ethicists that serious harm is the appropriate moral concept for triggering state intervention’ footnote on p. 44 (citing McDougall, R., & Notini, L., 2014 and other sources—that themselves cite McDougall, R., & Notini, L., 2014). Winters, J. (2018). When parents refuse: Resolving entrenched disagreements between parents and clinicians in situations of uncertainty and complexity. *American Journal of Bioethics*, *18*(8), 20–31. ‘While the debate is ongoing in this area … the review concludes, “There is substantial consensus among ethicists …’” (p. 23) (citing McDougall, R., & Notini, L., 2014). [III] *Medicine*: Isaacs, D., Preisz, A., Britton, P. N., Kilham, H. A., Robinson, P. D., & Farrow, G. (2019). Does asplenia make some immunisations obligatory? *Journal of Paediatrics and Child Health*, *55*, 499–501. ‘A systematic review … found a substantial consensus among ethicists’ (p. 501) (citing McDougall, R., & Notini, L., 2014).

72 Such evidence both pre‐ and post‐dates McDougall & Notini, op. cit. note 71, for example Pope, T. M. (2011). The best interest standard: Both guide and limit to medical decision making on behalf of incapacitated patients. *Journal of Clinical Ethics*, *22*(2), 134–138, and the articles and responses in an issue of *American Journal of Bioethics*, 2018, *18*(8)

73 We would stress that this is not intended as criticism of McDougall & Notini, op. cit. note 71. We only aim to illustrate how the label of systematic review might lead to consumers of the review to draw conclusions from it that they should not.

74 McDougall, R. (2015). Reviewing literature in bioethics research: Increasing rigour in non‐systematic reviews*. Bioethics*, *29*(7), 523–528; Parsons, J. A., & Johal, H. K. (2021). In defence of the bioethics scoping review: Largely systematic literature reviewing with broad utility. *Bioethics*, E‐pub ahead of print— 30 December 2021. https://doi.org/10.1111/bioe.12991

75 Lerner & Tetlock, op. cit. note 38; Perkins, D. N., Farady, M., & Bushey, B. (1991). Everyday reasoning and the roots of intelligence. In J. F. Voss, D. N. Perkins & J. W. Segal (Eds.), *Informal reasoning and education* (pp. 83–105). Lawrence Erlbaum; Peters, U. (2019). Implicit bias, ideological bias, and epistemic risks in philosophy*. Mind & Language*, *34*, 393–419.

76 Shapin, S., & Schaffer, S. (1985). *Leviathan and the air‐pump: Hobbes, Boyle and the experimental life*. Princeton University Press.

77 Harrington, J. (2017). *Towards a rhetoric of medical law*. Routledge, pp. 90–139.

78 Kant, I. (1998). *Critique of pure reason*, P. Guyer & A. W. Wood, Trans. Cambridge University Press, p. 109, B xiv.

